# Development of a dose verification system for Vero4DRT using Monte Carlo method

**DOI:** 10.1120/jacmp.v15i6.4961

**Published:** 2014-11-08

**Authors:** Yoshitomo Ishihara, Akira Sawada, Mitsuhiro Nakamura, Yuki Miyabe, Hiroaki Tanabe, Shuji Kaneko, Kenji Takayama, Takashi Mizowaki, Masaki Kokubo, Masahiro Hiraoka

**Affiliations:** ^1^ Department of Radiation Oncology and Image‐applied Therapy Graduate School of Medicine, Kyoto University Kyoto Japan; ^2^ Department of Radiological Technology Faculty of Medical Science, Kyoto College of Medical Science Nantan Japan; ^3^ Division of Radiation Oncology Institute of Biomedical Research and Innovation Kobe Japan; ^4^ Department of Radiation Oncology Kobe City Medical Center General Hospital Kobe Japan

**Keywords:** Monte Carlo, multileaf collimator, Vero4DRT, intensity‐modulated radiotherapy

## Abstract

Vero4DRT is an innovative image‐guided radiotherapy system employing a C‐band X‐ray head with gimbal mechanics. The purposes of this study were to propose specific MC models of the linac head and multileaf collimator (MLC) for the Vero4DRT and to verify their accuracy. For a 6 MV photon beam delivered by the Vero4DRT, a simulation code was implemented using EGSnrc. The linac head model and the MLC model were simulated based on its specification. Next, the percent depth dose (PDD) and beam profiles at depths of 15, 100, and 200 mm were simulated under source‐to‐surface distance of 900 and 1000 mm. Field size was set to $150\times 150\;{\text{mm}}^{2}$ at a depth of 100 mm. Each of the simulated dosimetric metrics was then compared with the corresponding measurements by a 0.125 cc ionization chamber. After that, intra‐ and interleaf leakage, tongue‐and‐groove, and rounded‐leaf profiles were simulated for the static MLC model. Meanwhile, film measurements were performed using EDR2 films under similar conditions to simulation. The measurement for the rounded‐leaf profile was performed using the water phantom and the ionization chamber. The leaf physical density and abutting leaf gap were adjusted to obtain good agreement between the simulated intra‐ and interleaf leakage profiles and measurements. For the MLC model in step‐and‐shoot cases, a pyramid and a prostate IMRT field were simulated, while film measurements were performed using EDR2. For the linac head, exclusive of MLC, the difference in PDD was <1.0% after the buildup region. The simulated beam profiles agreed to within 1.3% at each depth. The MLC model has been shown to reproduce dose measurements within 2.5% for static tests. The MLC is made of tungsten alloy with a purity of 95%. The leaf gap of 0.015 cm and the MLC physical density of $18.0\;g/\;{\text{cm}}^{3}$, which provided the best agreement between the simulated and measured leaf leakage, were assigned to our MC model. As a result, the simulated step‐and‐shoot IMRT dose distributions agreed with the film measurements to within 3.3%, with exception of the penumbra region. We have developed specific MC models of the linac head and the MLC in the Vero4DRT system. The results have demonstrated that our MC models have high accuracy.

PACS numbers: 87.55.K‐, 87.56.nk, 87.56.bd

## I. INTRODUCTION

A four‐dimensional, image‐guided radiotherapy system, Vero4DRT (MHI‐TM2000), was newly developed by Mitsubishi Heavy Industries, Ltd., Japan (MHI) in collaboration with Kyoto University and the Institute of Biomedical Research and Innovation (IBRI). The system has a gimbaled X‐ray head composed of a compact 6 MV linac with a C‐band klystron‐based accelerator, a fixed collimator, and a unique multileaf collimator (MLC).^1^, ^2^, ^3^, ^4^ The source‐to‐axis distance (SAD) is 1000 mm. In addition, electronic portal imaging devices and two sets of kilovoltage (kV) X‐ray tubes and flat‐panel detectors acquiring cone‐beam computed tomography and fluoroscopy are mounted on a rigid O‐ring–shaped gantry. The gimbaled X‐ray head enables its swing function to perform dynamic tumor‐tracking irradiation for a moving target using real‐time imaging and real‐time active beam adaptation.^(1)^

We have been developing an integrated Monte Carlo (MC) dose calculation system as a routine tool for verification of four‐dimensional dose calculation.^5^, ^6^, ^7^ In the past decade, applications of MC simulation in radiation therapy treatment planning and dosimetry have made great progress.^5^, ^6^, ^7^, ^8^, ^9^, ^10^, ^11^, ^12^, ^13^ It is now generally well accepted that MC is the most accurate dose calculation method because it can precisely model realistic radiation transport through a linac head, MLC, and patient anatomy.^14^, ^15^ MC simulations of radiotherapy beams require a detailed description of the geometry and materials of linac components contributing to production of the clinical radiation beams. Therefore, application of specification data, such as geometries of the linac head and the MLC from manufacturers, is of great importance.

Commissioning of MC simulation is generally performed using the following steps. First, the linac head, exclusive of MLC, is modeled against measurement data as a patient‐independent component. Next, the patient‐dependent MLC model is compared with several measurement data using well‐commissioned phase space data (PSD) from the linac head.^16^, ^17^, ^18^, ^19^, ^20^, ^21^, ^22^, ^23^

The MC‐based linac head model is verified by comparison between simulated and measured beam profiles and percent depth dose (PDD) profiles for rectangular fields with a variety of field sizes. Several researchers have reported that these rectangular fields were formed using specifically shaped collimators (e.g., CyberKnife^(16)^) or variable jaws (e.g., Varian,^17^, ^18^, ^19^ Elekta,^20^, ^21^, ^22^ and TomoTherapy^(23)^). However, the secondary collimator in the Vero4DRT is of a fixed type and, therefore, rectangular fields are formed using only MLC.^1^, ^4^

The purposes of this study were to develop specific MC models of a C‐band linac head with a fixed collimator and of a unique MLC in the Vero4DRT, and to verify specific MC models' accuracy.

## II. MATERIALS AND METHODS

### A. Vero4DRT treatment unit

**Figure 1:**
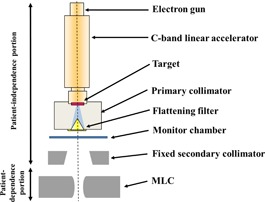
Geometric schema of the X‐ray head and MLC components for Vero4DRT system. The linac head was comprised of an electron gun, C‐band linear accelerator, a target, a primary collimator, a flattening filter, a monitor chamber, and a fixed secondary collimator. Modified from Mitsuhiro Nakamura et al.^(4)^

The linac head is composed of a compact C‐band 6 MV accelerator tube, a target, a flattening filter, a primary collimator, a fixed secondary collimator, and an MLC. The MLC is positioned just below the fixed secondary collimator (Fig. 1).

**Figure 2:**
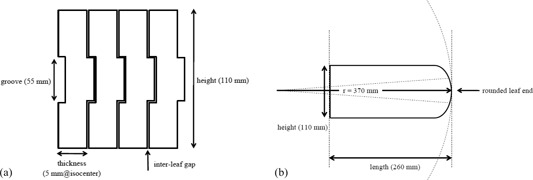
Schematic drawings of the MLC: (a) front and (b) side views. Modified from Mitsuhiro Nakamura et al.^(4)^

The MLC is made of tungsten alloy with a purity of 95%. It is of single‐focus type with 30 pairs of 5 mm wide leaves at the isocenter and covers a maximum field size of $150\times 150\;{\text{mm}}^{2}$.^(2)^ The direction of the MLC leaf travel is along lateral direction at home position. Interleaf leakage is minimized by an interlocking tongue‐and‐groove (T&G) arrangement. The groove part is 55 mm in height (Fig. 2(a)). The overall leaf height and the length are 110 mm and 260 mm, respectively (Fig. 2(b)). Each leaf end is rounded with a radius of curvature of 370 mm. The distance from the photon source to the lower edge of the MLC leaves is 500 mm. The distance of over‐travel of each leaf across the isocenter is 77.5 mm.^1^, ^4^

### B. Monte Carlo simulation parameters

The EGSnrc/BEAMnrc and EGSnrc/DOSXYZnrc codes^24^, ^25^ were used to simulate a 6 MV photon beam delivered by the Vero4DRT system. The detailed geometries of the linac head and the MLC were provided by MHI. Each simulation described in the Material & Methods sections C to E below was performed using parallel processing on a cluster of 2.6 GHz Intel Xeon E5 processors at the supercomputer of Academic Center for Computing and Media Studies, Kyoto University.^(26)^ The statistical uncertainty for each calculated voxel was within 1.0% beyond the depth of the maximum dose in the radiation field. The number of recycling of PSD was determined by automatic recycle function for all cases. The photon cutoff energy (PCUT) was set to 0.01 MeV; the electron cutoff energy (ECUT) was set to 0.521 MeV for all simulations.

### C. Verification of the linac head model

The characteristics of the incident electron beam are not sufficiently specified by the manufacturers. Many researchers have achieved good agreement with measurements using Gaussian‐shaped electron beam models.^27^, ^28^, ^29^

In this study, the mean energy of the incident electron beam on the target and the full width at half maximum (FWHM) of the radial intensity distribution were chosen to match the measurement results based upon previously published works.^27^, ^28^, ^29^ The mean energy of the incident electron beam varied from 5.5 to 7.0 MeV in steps of 0.1 MeV. Both the distributions of the energy and the intensity of the incident electron beam were expressed as Gaussian. The FWHM for the energy distribution was consistently set at 3% of the mean energy, while the FWHM for radial intensity distributions was set between 0.6 mm and 1.4 mm in step of 0.1 mm. For MC simulation, $8.0\times 10^{9}$ electron histories were simulated. The PSD was scored at a plane just proximal the fixed secondary collimator. The approximate $3.5\times 10^{7}$ particle data for an open field of $150\times 150\;{\text{mm}}^{2}$ were saved in a PSD file (2.9 GB).

Next, the PDDs and the beam profiles at depths of 15, 100, and 200 mm were computed under a source‐to‐surface distance (SSD) of 900 mm and 1000 mm with a voxel size of $0.5\times 0.5\times 0.5\;{\text{cm}}^{3}$. The dose at each point along the PDD profiles and the beam profiles were normalized to that at a depth of 15 mm for an open field of $150\times 150\;{\text{mm}}^{2}$. The simulation result was then compared with the corresponding measurement using a water phantom and a 0.125 cc ionization chamber (model 31010; PTW, Freiburg, Germany).

Generally, the MC‐based linac head is verified using PDDs and beam profiles for several rectangular fields formed by specifically shaped collimators or variable jaws.^16^, ^17^, ^18^, ^19^, ^20^, ^21^, ^22^, ^23^ On the other hand, the secondary collimator in the Vero4DRT is of a fixed type and the field is formed using the MLC only. However, the beam profiles for fields obtained by the MLC without its verification are not appropriate, leading to a lack of dose data for verification of the MC linac head model. To compensate for a lack of several rectangular fields dose data, PDD profiles at several SSDs and beam profiles at several depths were acquired in this study.

### D. Verification of the static MLC model

The results of EGSnrc/BEAMnrc simulations of several types of MLCs, such as ModuLeaf MLC,^(21)^ BrainLAB microMLC,^(30)^ Millennium 120,^17^, ^18^ and HD120MLC,^(19)^ have been reported. BEAMnrc provides a series of component modules (CM) for modeling various types of MLC with ease. The MLC for the Vero4DRT was then fully modeled using one of the CM “VARMLC”.^(31)^

The relevant simulation parameters such as the abutting leaf gap, the MLC density, and Zmin were chosen to minimize differences between simulated and measured data.

For the MLC model, three static tests were simulated, employing well‐commissioned PSD from the linac head model: 1) intra and interleaf leakage; 2) tongue‐and‐groove (T&G) effect; and 3) rounded leaf end profiles.

On the other hand, film measurements were performed using EDR2 films (Eastman Kodak Company, Rochester, NY) and water‐equivalent phantoms under the same conditions as the corresponding simulation for intra‐ and interleaf leakage and T&G tests, respectively. A film calibration dataset was acquired by placing a film at a 100 mm depth for SAD of 1000 mm. The film was irradiated perpendicular to the beam axis with a field size of $50\times 50\;{\text{mm}}^{2}$ at the home position. The dose delivered at 100 mm depth was calculated by combining the delivered MU, tissue maximum ratio (TMR), and output factor (OF). Both TMR and OF were measured using a 0.6 cc ionization chamber (TN30013; PTW) annually calibrated by the National Institute of Radiological Science. Subsequently, we delivered a small square pattern with 10 incremental dose levels to separate films, respectively. The corresponding absolute dose was measured using the calibrated ion chamber. Then, all the films for those square patterns were scanned using a flatbed scanner (ES‐10000G; Epson Corp., Nagano, Japan) with a resolution of 150 dpi in 16‐bit grayscale. Next, the scanner number value was associated with the corresponding measured dose in order to acquire the film calibration curve.^(4)^ Using the film calibration curve, all the films irradiated in this study were scanned using the same scanner, and the scanner number values were converted to the absolute dose and were analyzed using a DD‐System (R‐TECH Inc., Tokyo, Japan).

For the rounded‐leaf effect, the measurement was performed using water phantom, IBA CC01 ionization chamber (Iba Dosimetry, Schwarzenbruck, Germany), and the 0.125 cc ionization chamber.

#### D.1 Intra‐ and interleaf leakage

**Figure 3:**
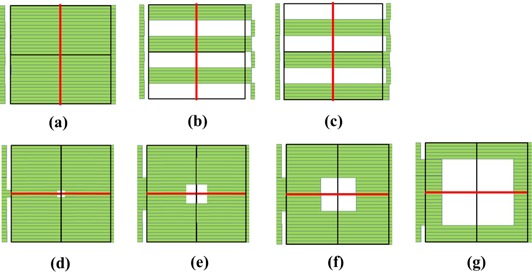
MLC patterns of the static tests for the MLC model. Dose profiles were created along red lines: intra‐ and interleaf leakage pattern (a)‐(g). T&G profiles were simulated by adding (b) with (c). Rounded‐leaf profiles were calculated for (d) $10\times 10\;{\text{mm}}^{2}$, (e) $30\times 30\;{\text{mm}}^{2}$, (f) $50\times 50\;{\text{mm}}^{2}$, and (g) $100\times 100\;{\text{mm}}^{2}$.

The intra‐ and interleaf leakage test was performed to evaluate the transmission properties of the MLC and the MLC shape of the longitudinal direction. Figure 3(a) illustrates a MLC pattern for the intra‐ and interleaf leakage test.

The MLCs were parked behind the fixed secondary collimator. The measurement for the intra‐ and interleaf leakage test was performed by irradiating a film with 15,000 MU at a depth of 100 mm for a SAD of 1000 mm at the home position. After leaf leakage measurement, another fresh film was placed at the same position and then irradiated with 150 MU for an open field of $150\times 150\;{\text{mm}}^{2}$. The intra‐ and interleaf leakage dose along the direction perpendicular to MLC travel was determined as the ratio of $\frac{\text{dose}}{\text{MU}}$ at each point with the MLC fully closed to $\frac{\text{dose}}{\text{MU}}$ at the isocenter with the MLC fully opened.

MC leaf leakage was simulated under the same conditions as the measurement with a voxel size of $0.2\times 0.2\times 0.2\;{\text{cm}}^{3}$.

#### D.2 Tongue‐and‐groove effect

Fields with irregularly formed patterns were simulated to verify the T&G effect for our MLC model and to evaluate the MLC shape along the longitudinal direction. A field of $150\times 150\;{\text{mm}}^{2}$ was formed by the MLC, and groups of five leaf pairs were placed alternately in and out of the field in the T&G effect test (Figs. 3(b), (c)). Next, 150 MU were delivered to the field with a film placed at a depth of 100 mm for an SAD of 1000 mm at the home position. Subsequently, 150 MU were delivered to the field formed by switching alternating leaf positions. Measured doses were then normalized to the dose at the isocenter for the fully open field.

MC T&G profiles with a voxel size of $0.2\times 0.2\times 0.2\;{\text{cm}}^{3}$ were computed under the same conditions as described above.

#### D.3 Rounded leaf end profiles

Fields with nominal sizes of 10×10, 30×30, 50×50, and $100\times 100\;{\text{mm}}^{2}$ were formed by the MLC, respectively (Figs. 3(d)‐(g)). Fields smaller than $30\times 30\;{\text{mm}}^{2}$ were measured using IBA CC01 ionization chamber at a depth of 100 mm for an SAD of 1000 mm. Fields larger than $50\times 50\;{\text{mm}}^{2}$ were measured using the water phantom and the 0.125 cc ionization chamber at a depth of 100 mm for an SAD of 1000 mm. The measured doses were normalized to the dose at the isocenter for each field. This test was performed to evaluate the MLC shape along the lateral direction. And, the accuracy of the position and size of fields formed by the modeled rounded leaves was verified.

After verification of the static MLC model, the linac head model combined with the MLC model was verified by computing PDDs for 30×30,50×50, and $100\times 100\;{\text{mm}}^{2}$ with a voxel resolution of $0.5\times 0.5\times 0.5\;{\text{cm}}^{3}$. Each PDD was normalized to the corresponding dose at a depth of 15 mm for each field. Each was then compared with the corresponding measured dose profile.

### E. Verification of the step‐and‐shoot MLC model

For the MLC model in step‐and‐shoot irradiations, both a pyramid intensity distribution case and a prostate IMRT case were simulated using a water‐equivalent phantom with a voxel size of $0.2\times 0.2\times 0.2\;{\text{cm}}^{3}$. Seven segments for a field of $150\times 150\;{\text{mm}}^{2}$ were applied for the pyramid intensity distribution case, while a leaf sequence file with 32 segments was created using iPlan RT (BrainLAB, Feldkirchen, Germany) for the prostate IMRT case. The step‐and‐shoot motion of the MLC leaves was simulated by sampling the leaf positions for each incident history using a cumulative probability distribution function of each leaf position, which can be created from a correlation between the fractional number of MU and the corresponding leaf positions specified in the .mlc leaf sequence file. A similar method was used by Liu et al.^(32)^ for the DMLCQ component module.

Meanwhile, film measurements were performed at a depth of 100 mm in the water‐equivalent phantom. The doses were normalized to the dose at a depth of 100 mm along the central axis. The difference between the simulated and measured dose was calculated along the in‐plane and cross‐plane directions, respectively.

## III. RESULTS

### A. Linac head model verification

**Figure 4:**
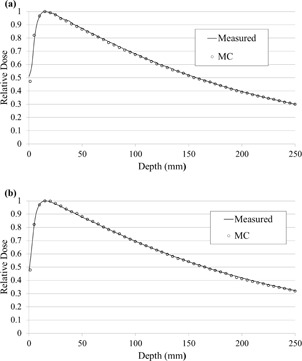
PDD profiles of the simulated doses with a voxel resolution of $0.5\times 0.5\times 0.5\;{\text{cm}}^{3}$ and measured doses: (a): SSD=900mm, and (b): SSD=1000mm.

In the linac head model verification, MC statistical uncertainty was controlled below 1% for all irradiated fields. Figure 4 shows comparisons of measured and simulated PDDs for an open field of $150\times 150\;{\text{mm}}^{2}$ in the water phantom at different SSDs. The simulated PDD beyond the buildup point showed an agreement of with 1.0%.

**Figure 5:**
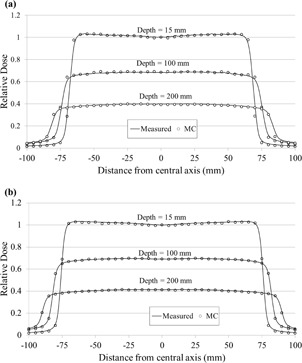
Beam profiles as a function of distance from the central axis of simulated doses with a voxel resolution of $0.5\times 0.5\times 0.5\;{\text{cm}}^{3}$, and measured doses at depths of 15, 100, and 200 mm, respectively: (a) SSD=900mm; (b) SSD=1000mm.

Figure 5 depicts the simulated and measured beam profiles for fully open fields in the water phantom at depths of 15, 100, and 200 mm with different SSDs. The simulated beam profiles, exclusive of the penumbra region, agreed within 1.3% at each depth and SSD. The differences in the field size calculated from each measured and simulated profile were within 1.0 mm at each depth.

In the commissioning of photon beam PSD created from the Vero4DRT, the best agreement between the MC simulation and the measurement was obtained for the mean energy of an incident electron beam of 6.7 MeV and a Gaussian intensity profile with an FWHM of 1.0 mm.

The above results have demonstrated that our MC model of the linac head with fixed collimators on the Vero4DRT system could be achieved with high accuracy.

### B. MLC model verification

In the MLC model verification, MC statistical uncertainty was controlled below 1% for all irradiated fields.

#### B.1 Static MLC model

##### B.1.1 Intra‐ and interleaf leakage

**Figure 6:**
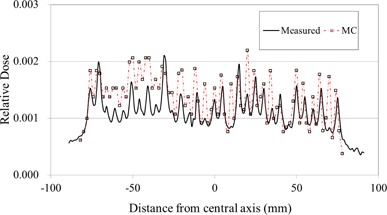
Intra‐ and interleaf leakage profiles as a function of distance from the central axis of simulated doses with a voxel resolution of $0.2\times 0.2\times 0.2\;{\text{cm}}^{3}$, and measured doses.

Figure 6 shows a comparison of the simulated and measured leaf leakage along the lateral axis. Each inter‐ and intraleaf leakage profile was normalized to the corresponding dose at the isocenter for the fully open field, respectively. The leaf gap and physical density of the MLC were chosen to minimize the difference between the simulated and measured doses. As such, the upper edge of the MLC, Zmin, was set to be 38.9 cm below the target in the linac head; the interleaf gap was set to 0.015 cm. The MLC physical density of $18.0\;g/{\text{cm}}^{3}$ provided the best agreement between the simulated and the measured leaf leakage. From the MLC simulation result, the interleaf leakage was 0.22%, whereas the intraleaf leakage was <0.08% and the average leaf leakage for the entire field was 0.13%. In the measurement, the interleaf, intraleaf, and average leaf leakage values were 0.21%, <0.12%, and 0.11%, respectively. These results demonstrate that our MC model has the capability to simulate leaf leakage with high accuracy.

##### B.1.2 Tongue‐and‐groove effect

**Figure 7:**
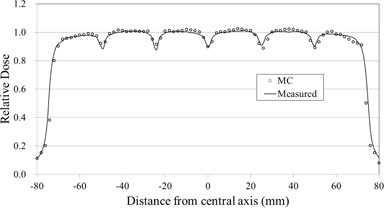
T&G profiles as a function of distance from the central axis of simulated doses with a voxel resolution of $0.2\times 0.2\times 0.2\;{\text{cm}}^{3}$, and measured doses.

Figure 7 shows a comparison of simulated and measured T&G profiles along the longitudinal axis. The simulated and measured profiles agreed with <2.5% for most points. The T&G underdosage effect was 10.1% in the simulation and 10.7% in the measurement.

##### B.1.3 Rounded leaf end profiles

**Figure 8:**
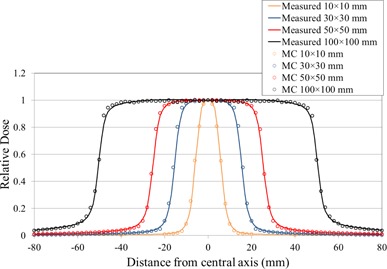
Rounded‐leaf profiles as a function of distance from the central axis for simulated and measured doses. Fields were formed by the MLC for nominal field sizes of 10×10,30×30,50×50, and $100\times 100\;{\text{mm}}^{2}$.

Figure 8 shows the comparisons between the simulated and measured longitudinal dose profiles at a depth of 100 mm in water with an SSD of 900 mm for fields of 10×10,30×30,50×50, and $100\times 100\;{\text{mm}}^{2}$, respectively. The difference between the simulated and the measured doses agreed within 1.5%, except for the penumbra region. Agreement in the 80%‐20% penumbra widths was better than 1.0 mm for all the fields that were compared. In the penumbra region of all the fields, distance‐to‐agreement is less than 0.5 mm.

**Figure 9:**
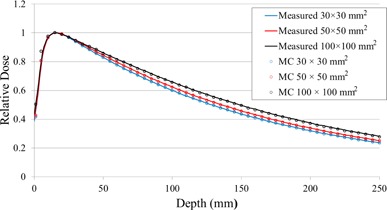
PDD profiles of simulated doses with several field sizes, and measured doses.

Figure 9 shows simulated and measured PDDs for fields of 30×30,50×50, and $100\times 100\;{\text{mm}}^{2}$, respectively. The difference between the simulated and measured PDDs was within 1.6% beyond the buildup region for each field.

The above results demonstrate that our MLC model using the VARMLC component module is feasible for simulation of a dose effect based on a specific MLC shape in the Vero4DRT system.

#### B.2 Step‐and‐shoot model

**Figure 10:**
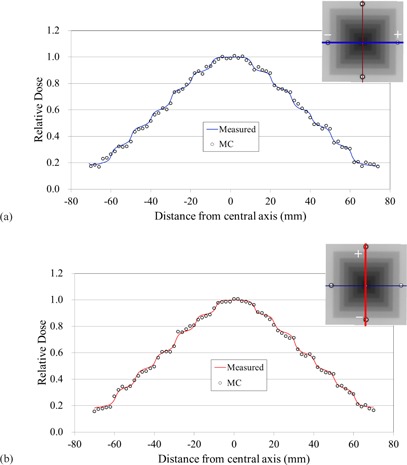
Measured and simulated profiles of a pyramid‐intensity distribution case. Dose was computed with a voxel resolution of $0.2\times 0.2\times 0.2\;{\text{cm}}^{3}$: (a) in‐plane direction; (b) cross‐plane direction.

Figure 10 shows a comparison of simulated and measured dose profiles for a pyramid intensity distribution case delivered with a step‐and‐shoot technique. Figures 10(a) and (b) represent dose profiles along the in‐plane and cross‐plane directions, respectively. The difference between the simulated and the measured doses was within 2.5% between 20% and 100% dose area on both profiles.

**Figure 11:**
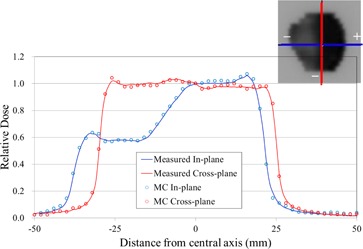
Measured and simulated profiles of a step‐and‐shoot IMRT case. Dose was computed with a voxel resolution of $0.2\times 0.2\times 0.2\;{\text{cm}}^{3}$. Blue line = in‐plane direction; red line = cross‐plane direction.

Figure 11 shows an example of the simulated and the measured dose profiles along the in‐plane and the cross‐plane directions for a step‐and‐shoot IMRT case. The simulated and measured profiles agreed within 3.3%.

Our MC system could simulate dose gradients in the step‐and‐shoot case with high accuracy; however, the difference between the simulated and measured doses for the step‐and‐shoot test was slightly larger than for the leaf‐leakage and T&G film tests for the static field. This may have been due to the uncertainty of EDR2 film measurements for the low‐dose range.^10^, ^33^, ^34^ The MU of the step‐and‐shoot case was less than half that of the leaf leakage and T&G film tests. Several researchers reported on similar results in their commissioning process.^15^, ^32^

## IV. DISCUSSION

The linac head model, with a compact C‐band accelerator and a newly designed MLC for the Vero4DRT system, was simulated using EGSnrc/BEAMnrc and EGSnrc/DOSXYZnrc codes with high accuracy.

In the linac head model, exclusive of MLC, good agreement between the MC simulations and measurements using an ionization chamber and water phantom dosimetry was obtained. The difference in PDDs was <1.0% beyond the buildup region. The simulated beam profiles agreed to within 1.3% for all depths and SSDs.

The MC MLC model has been shown to reproduce dose measurements within 2.5% for static tests exclusive of the penumbra. The simulated step‐and‐shoot IMRT dose distributions agreed with the dose distributions from film measurements within 3.3% with exception for the penumbra region.

## V. CONCLUSIONS

We have developed specific MC models of a C‐band linac head with a fixed collimator and of a unique MLC in the Vero4DRT. The results have demonstrated that our MC models have high accuracy.

## ACKNOWLEDGMENTS

The authors would like to thank all radiotherapy technicians at IBRI for data acquisition. We would like to express our appreciation to the entire technical staff of MHI for providing detailed information on the MLC. This work was supported by JSPS KAKENHI Grant Numbers 25253078 and 60709351.
